# Supplementary material for: Influence of the Addition of Extruded Endogenous Tartary Buckwheat Starch on Processing and Quality of Gluten-Free Noodles
Source: Foods. 2021 Nov 4;10(11):2693. doi: 10.3390/foods10112693 (PMC8618275; doi:10.3390/foods10112693)
Supplement: Supplementary file 1 [file foods-10-02693-s001.zip › foods-1449888-supplementary.pdf]

Supplementary Materials

Supplementary Table S1 Drying procedure for gluten-free *Tartary* buckwheat noodles

Steps	Time (min)	Temperature (°C)	Humidity (%)	Cycle frequency (Hz)	Stirring Frequency (Hz)	Remove-water Frequency (Hz)
1	5	35	85	35	25	15
2	25	35	85	35	25	15
3	5	40	80	35	25	15
4	30	40	80	35	25	15
5	5	45	75	35	25	15
6	50	45	75	35	25	15
7	5	40	70	35	25	15
8	15	40	70	35	25	15
9	10	30	60	35	25	15
10	20	30	60	35	25	15

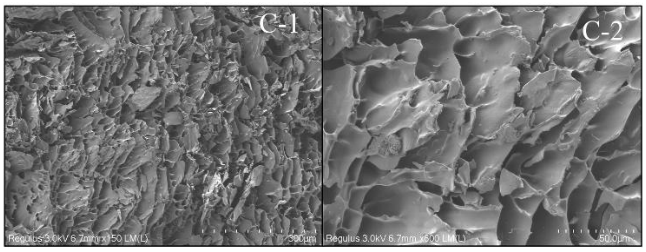

Supplementary Figure S1 Microstructure of 15% extruded starch gel.
